# Methods of Resource Scheduling Based on Optimized Fuzzy Clustering in Fog Computing

**DOI:** 10.3390/s19092122

**Published:** 2019-05-08

**Authors:** Guangshun Li, Yuncui Liu, Junhua Wu, Dandan Lin, Shuaishuai Zhao

**Affiliations:** School of Information Science and Engineering, Qufu Normal University, Rizhao 276800, China; Guangshunli@qfnu.edu.cn (G.L.); 15163315741@163.com (Y.L.); 15725040625@163.com (D.L.); zhaoshuaishuaiys@163.com (S.Z.)

**Keywords:** fog computing, fuzzy c-means algorithm, particle swarm optimization, resource clustering, resource scheduling

## Abstract

Cloud computing technology is widely used at present. However, cloud computing servers are far from terminal users, which may lead to high service request delays and low user satisfaction. As a new computing architecture, fog computing is an extension of cloud computing that can effectively solve the aforementioned problems. Resource scheduling is one of the key technologies in fog computing. We propose a resource scheduling method for fog computing in this paper. First, we standardize and normalize the resource attributes. Second, we combine the methods of fuzzy clustering with particle swarm optimization to divide the resources, and the scale of the resource search is reduced. Finally, we propose a new resource scheduling algorithm based on optimized fuzzy clustering. The experimental results show that our method can improve user satisfaction and the efficiency of resource scheduling.

## 1. Introduction

With the rapid development of technology related to the Internet of Things (IoT) [1], an increasing amount of data is being transmitted through the Internet. Cloud computing, as a distributed computing model, can store and process the massive data generated by the IoT and provide terminal users with reliable services [2,3]. However, smart devices consume a large amount of network bandwidth and aggravate the burden of cloud data centers [4,5], such that some delay-sensitive services in the IoT cannot be responded to and processed quickly. In addition, many requirements of edge devices, such as real-timeliness, mobility, location awareness, etc., cannot be met. A new computing paradigm called fog computing was proposed by Cisco in 2012, which extends the traditional cloud computing paradigm to the edge of the network [6].

As a virtualized platform, fog computing is located between the IoT devices and cloud servers, and provides computing and storage services at the edge of the Internet. As a distributed architecture, fog computing consists of devices with weak performance and wide distribution that are closer to terminal users. When a service request is raised by the terminal device in the network, first, data filtering, preprocessing, and analysis are performed in fog computing. The processed data are then transmitted to the cloud computing system, which can reduce the burden on the cloud data center.

With the development of IoT technologies, the number of mobile devices located at the edge of the network increases quickly [7]. Therefore, massive data must be stored and processed to meet the various user requests. Cloud computing has good data storage and processing capabilities, while central servers are far from the end users, which may result in large delays. Especially for delay-sensitive applications, the quality of service will be greatly reduced. Therefore, fog computing is more suitable for terminals. In addition, due to the dynamicity and uncertainty of the resources as well as the high variability and unpredictability of the fog environment, resource scheduling is a key technology that must be urgently resolved. In fog computing, resource scheduling faces the problem of massive data. A fuzzy clustering algorithm is an effective resource classification method. In addition, when resource attributes cannot be accurately described, fuzzy theory provides different effective means to solve the uncertain problems in the real world. Fuzzy c-means clustering is an effective algorithm, but the random selection of center points makes the iterative process easily fall into the local optimal solution. Therefore, we combine the fuzzy clustering algorithm with particle swarm optimization (PSO) to achieve global optimization. In this paper, we chose the improved fuzzy clustering algorithm to solve the resource scheduling problem in fog computing.

We begin by proposing a fog computing framework. Then, we standardize and normalize the resource attributes, and we combine the fuzzy clustering with particle swarm optimization to divide resources, thereby reducing the scale of the resource search. In addition, according to the weight matching, we match the resources and tasks to get the final resource scheduling results. Finally, based on simulations and numerical results, we conclude that our method can improve user satisfaction.

The remainder of this paper is organized as follows: the related work is discussed in the Section 2, the fog computing architecture is described in the Section 3, the resource scheduling problem in fog computing is described in the Section 4, the resource scheduling algorithms are proposed in the Section 5, the experimental results are analyzed in the Section 6, and Section 7 summarizes the full text.

## 2. Related Work

Resource scheduling in cloud computing is an NP-hard problem [8]. To solve this problem, various resource scheduling algorithms are proposed. Mittal et al. [9] proposed an optimal task scheduling algorithm in cloud computing, which adapted the advantages of various algorithms according to the situation, such as Min–Min, Max–Min, and RASA. It also considered the distribution and scalability of cloud resources. This method achieved a lower makespan compared with other existing algorithms. In addition, some artificial intelligence-based algorithms (particle swarm optimization algorithms, genetic algorithms, etc.) were also proposed [10,11]. However, these algorithms dealt with the entirety of the resources, thus the costs of resource selection were large. Therefore, resource clustering methods have been proposed to reduce the range of resource selection. Li et al. [12] proposed a two-level cloud task scheduling algorithm based on fuzzy clustering to reduce the makespan. Wu et al. [13] proposed an improved fuzzy clustering algorithm to reduce the number of matching requests in mobile cloud computing. The matching strategy was dynamically adjusted according to the matching scores and feedback training. Zhang et al. [14] proposed a scheduling optimization strategy of task and resource hybrid clustering based on fuzzy clustering to narrow the task scheduling scale and introduced an improved Bayesian classification algorithm that fast matches tasks and computers. Guo et al. [15] presented a workflow task scheduling algorithm based on the resources’ fuzzy clustering named FCBWTS. In this algorithm, the resource characteristics of cloud computing were considered, and the processing unit network is pretreated using the fuzzy clustering method, which reduced the costs for deciding which processor to use to execute the current task.

Fog computing extends the cloud computing model and provides services for local mobile devices. The structure and basic theory of fog computing are discussed. In addition, related research has been conducted on the resource management of fog computing. At present, the research on fog computing is still in the early stages.

In their study, Aazam et al. [16] proposed a service oriented resource management model, which can effectively manage resource for the IoTs in fog computing. Based on users’ behavior and the probability of future use, the model can allocate resources in advance. Su et al. [17] proposed a Steiner tree-based resource caching scheme. The generated Steiner tree was used to minimize the resource cache costs compared with the shortest path scheme. The results showed that this scheme can achieve better performance.

He et al. [18] combined fog computing with a software-defined network (SDN) in a vehicle networking environment and proposed an improved SDN-based constrained optimization particle swarm algorithm. The algorithm can effectively reduce the latency and promote the quality of service. Pham et al. [19] considered the task scheduling problem in a cloud-fog system and proposed a heuristic-based algorithm to achieve the balance between the maximum completion time and the monetary costs of cloud resources. Zeng et al. [20] considered a fog computing supported software-defined embedded system and studied three key issues of task scheduling. To improve user experience, these problems were formulated as a mixed-integer nonlinear programming problem, and the research proposed an efficient heuristic task scheduling strategy. Ni et al. [21] proposed a resource allocation strategy in fog computing based on Priced Timed Petri Nets (PTPNs). In addition, according to the characteristics of fog resources, the PTPN model of tasks was constructed. The proposed algorithm can achieve higher efficiency than static allocation strategies. Some of the above research study resource scheduling strategies combined with a specific network in fog computing. In addition, some studies used heuristic algorithms to implement resource scheduling. However, the above algorithms were designed with respect to overall resources. When the number of resources was large, there were a large selection of resources and a reduction in user satisfaction. Therefore, this paper considers a fuzzy clustering algorithm to cluster resources and reduce the scope of the resource search. In addition, because the fuzzy clustering algorithm is sensitive to the initial value and easy to fall into the local optimum, this paper combines it with the particle swarm algorithm to improve the defects of the clustering algorithm. The combination of these two algorithms has been reported in some existing literature. Kang et al. [22] presented a hybrid approach for text document clustering based on the fuzzy c-means and particle swarm optimization, which makes full use of the merits of both algorithms. Mekhmoukh et al. [23] presented an improved kernel possibilistic c-means (KPCM) algorithm and incorporated the spatial neighborhood information and particle swarm optimization algorithm for cluster centers and membership initialization. The improved algorithm was applied to solve image segmentation. Unlike these studies, our paper combines the two algorithms to solve the resource scheduling problem in fog computing.

## 3. Fog Computing Architecture

**Figure 1:**
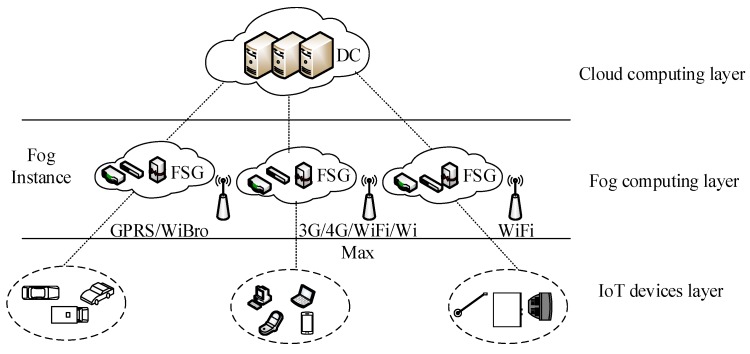
Fog computing architecture.

Cloud computing is centralized and composed of powerful servers, which can be extended to the edge of the network using fog computing. Fog computing consists of some weaker and more dispersed servers, which are closer to terminal users. In addition, fog computing can provide flexible resources to completely heterogeneous computing and storage requests in the IoT. Currently, the general fog computing architecture can be regarded as a three-tiered network structure, as shown in Figure 1.

The IoT device layer includes various types of devices, such as smart phones, tablet computers, smart vehicles, and different smart home devices. The IoT can sense the surrounding environment and collect data continuously through some sensor devices. In addition, this layer communicates with the fog computing layer through 3G, 4G, WiFi, and WiBro technologies, and then uploads the collected sensor data. The fog computing layer consists of many edge servers, including some heterogeneous devices, such as routers, switches, gateways, and access points. The fog computing layer can be seen as fog instances (FIs) [24]. Each fog instance contains a fog smart gateway (FSG) [25]. The FSG can implement heterogeneous network conversion and determine the type of data and timings to be processed in the fog. This layer can collect data and perform data preprocessing to filter out redundant or abnormal data and then upload them to the cloud. Moreover, fog computing is suitable for low-latency applications such as video streaming, augmented reality, and online games [26]. The cloud computing layer includes many powerful high-end servers; the cloud data center processes and stores large amounts of data, and enables efficient resource management by deploying virtualization technologies [27]. This level supports the long-term analysis of the uploaded data by the fog computing layer.

In the fog computing architecture that was mentioned above, fog computing is a microdata center paradigm that provides computing, storage, and network services between the cloud and the IoT. Fog computing has a data cache function that can predict user requests according to the current environment and actively cache the response data from the cloud server. Furthermore, it also stores the data that are uploaded by terminal users through passive caching. To a great extent, it reduces the transmission delays and the burden of cloud data centers. In addition, a fog computing application includes a client’s mobile device, one of potentially many fog nodes, and back-end cloud servers. Therefore, compared with cloud computing, it is difficult to complete task scheduling in fog computing [28]. The fog nodes analyze the task requests that are sent by terminal devices, divides them into several subtasks for processing, and selects the matching resources in the resource pool to meet the users’ requirements.

## 4. Description of Resource Scheduling Problem in Fog Computing

In most cases, resource scheduling can be seen as a task scheduling problem that allocates resources to users’ tasks. If the resource scheduling is performed in the cloud, large scale data transmission may consume a large amount of network bandwidth and add the burden of cloud data centers. In addition, resource scheduling in fog computing depends on the kind of user service request. Service requests include delay tolerance and delay sensitivity. The cloud computing is far away from the end users, which will generate a large transmission delay, but fog computing is close to the end users, which has the advantages of low delay and location awareness. Therefore, the delay sensitive user requests are usually processed in the fog computing layer, and the delay tolerant user requests are scheduled in the cloud computing layer. Therefore, this paper focuses on resource scheduling in fog computing. The specific differences between cloud-based scheduling and fog-based scheduling are shown in Table 1. Due to the diversity of user requests, fog computing will allocate resources according to the users’ needs. The goal of resource scheduling is to find the best matching resources for users to achieve the optimal scheduling goals, such as reducing the processing delay and improving resource utilization and quality of service (QoS).

**Figure 2:**
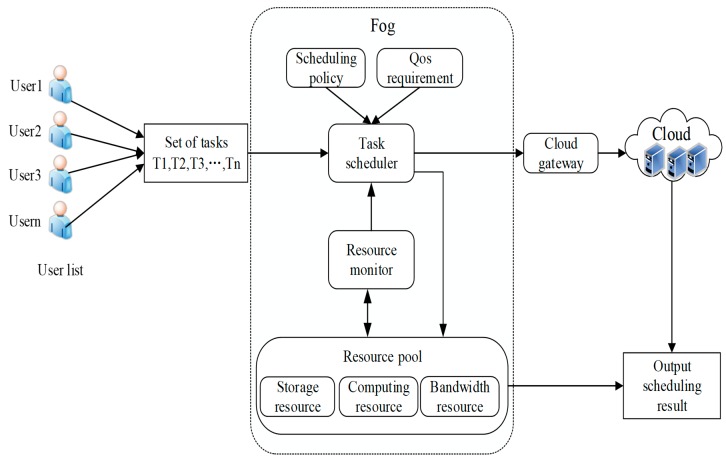
Fog computing resource scheduling process.

The fog computing resource scheduling process is shown in Figure 2. First, when users submit tasks, we can divide a larger task into many smaller subtasks. Then, these subtasks will be submitted to the task scheduler in the fog environment where the task scheduling strategy and QoS request play decisive roles in the task scheduler. The task scheduler collects the scheduling data from users, resource monitors and cloud gateways, and then assigns each task to the corresponding fog resource. Therefore, we propose a resource scheduling algorithm to find the optimal matching of tasks and resources. The resource monitor observes the fog resource pools, including storage resources, computing resources, and bandwidth resources. When the task requests of the terminal users that are to be processed exceed the computing capability of fog computing, these tasks may be submitted by cloud servers for further processing when necessary. Finally, according to a certain scheduling strategy, resources and user requests achieve the corresponding match, and the final scheduling results will be returned to users.

First, assume that there are n tasks and m resources, the task set is $T=\{t_{1},t_{2},t_{3},\cdots ,t_{n}\}$, the fog resources set is $R=\{r_{1},r_{2},r_{3},\cdots ,r_{m}\}$, and rational resource scheduling is completed according to a certain scheduling strategy. Among them, the task model and the resource model are represented as follows.

(1) Task model: the task set that is submitted by users includes n fog tasks. The *i*-th task is represented by $T_{i}$, and its features are described as a one-dimensional vector $T_{i}=\{t_{id},t_{len},t_{comp},t_{netw},t_{stor},t_{dat}\}$; where $t_{id}$ is the task number; $t_{len}$ is the task length; $t_{comp}$, $t_{netw}$, and $t_{stor}$ are the task’s computing power, bandwidth capacity, and storage capacity requirements for the resource, respectively; and $t_{dat}$ is the data that must be processed by the task.

(2) Resource model: in fog computing, the physical resources can be abstracted into virtual resources through virtualization technology. Assuming that there are m resources in the set of fog resources, the *j*-th resource is represented by $R_{j}$, and its features are described as a one-dimensional vector $R_{j}=\{r_{id},r_{comp},r_{netw,}r_{stor}\}$. Here, $r_{id}$ is the resource number; and $r_{comp}$, $r_{netw}$, and $r_{stor}$ are the computing power, bandwidth capacity, and storage capacity of the resource, respectively.

**Figure 3:**
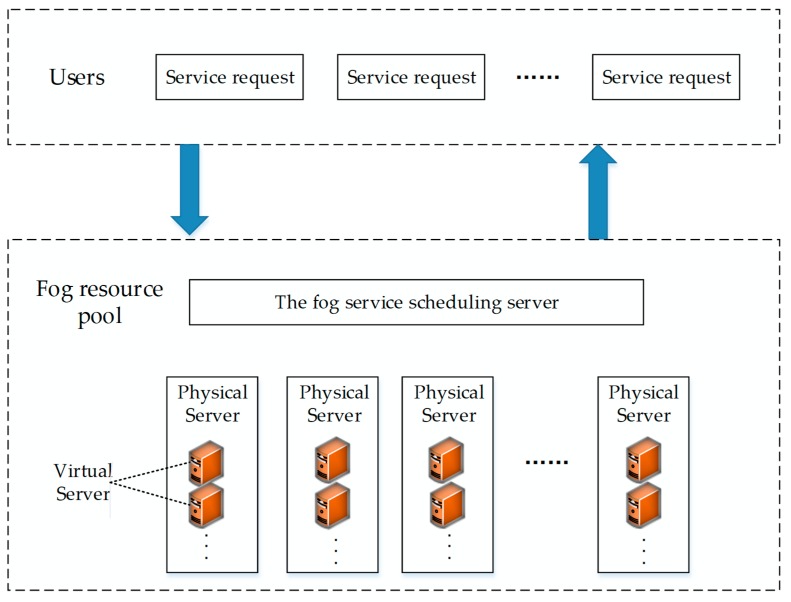
Fog computing resource scheduling network architecture.

To show the connections of the entities in the model, we provide a resource scheduling network architecture in fog computing (Figure 3).

In the scheduling process, the following constraints must be satisfied.

(1) The limitation of the computing nodes in the resource pool: the computing resources and storage space that are used by tasks must be within the scope of the fog computing nodes. Each computing node can handle one task at a time; however, all computing nodes can be executed in parallel.

(2) User task execution restriction: all tasks need to be assigned to specific fog computing nodes to be performed, and each task can only be executed on one fog computing node.

In this article, we mainly consider the QoS as an evaluation index, which is used to evaluate the effectiveness of resource scheduling in fog computing.

QoS: It is an important criterion for measuring service satisfaction. It is a key aspect that should be considered in resource scheduling. Every user has different resource requirements. To better serve various users, we need to improve the QoS.

## 5. Resource Scheduling Algorithms in Fog Computing

### 5.1. Fuzzy Clustering Algorithm with Particle Swarm Optimization (FCAP)

Based on the traditional Fuzzy C-Means (FCM) clustering algorithm [29] and particle swarm optimization (PSO) algorithm [30], we propose the FCAP algorithm to achieve resource scheduling in fog computing. The main idea is that we put the PSO algorithm in the FCM algorithm.

The FCM clustering algorithm determines the degree to which each sample point belongs to a certain cluster using a membership function. Let the cluster sample set be $X=\{x_{1},x_{2},x_{3}\cdots ,x_{n}\}\subset R^{d}$, where $x_{i}$ is the d dimension vector. We need to classify the sample set into c classes. Set the cluster center as $V=\{v_{1},v_{2},v_{3}\cdots ,v_{c}\}$, and define the degree to which the sample points belong to the *j*-th class as $\mu_{ij}$. In addition, the fuzzy matrix of the sample space X is $U=(\mu_{ij})$.

The FCM clustering algorithm can be expressed as the following objective function for the extremum problem:

(1) $$J=\min\sum_{i=1}^{n}\sum_{j=1}^{c}\mu_{ij}{}^{m}{\left\|x_{i}-v_{j}\right\|}^{2}$$

$$\begin{array}{ll} s.t. & \sum_{j=1}^{c}\mu_{ij}=1,\;\mu_{ij}\in [0,1],\\ & i=1,2,\cdots ,n,\;j=1,2,\cdots ,c. \end{array}$$

In Equation (1), $\mu_{ij}$ is the degree of belongingness of the *j*-th data point to the *i*-th cluster, $v_{j}$ is the *j*-th cluster, $\left\|x_{i}-v_{j}\right\|$ is the Euclidean distance from the sample points $x_{i}$ to the cluster center $v_{j}$, and m is the fuzzy index. In addition, U and V can be calculated as follows:

(2) $$v_{j}=\frac{\sum_{i=1}^{n}\mu_{ij}{}^{m}x_{i}}{\sum_{i=1}^{n}\mu_{ij}{}^{m}}$$

(3) $$\mu_{ij}=\frac{1}{\sum_{k=1}^{c}{\left(\frac{\left\|x_{i}-v_{j}\right\|}{\left\|x_{i}-v_{k}\right\|}\right)}^{\frac{2}{m-1}}}$$

The algorithm is a local optimization method, which seeks the optimal solution by climbing hills. Its idea is to maximize the similarity between objects partitioned into the same cluster and minimize the similarity between different clusters. The fuzzy algorithm is an improvement of the ordinary C-Means algorithm. The ordinary C-Means algorithm is hard for data partition, while FCM is a flexible fuzzy partition. The FCM algorithm determines the degree to which each sample point belongs to a certain cluster using a membership function. There are no threshold values in the algorithm. The idea of soft partitioning adopted by the membership matrix, and finally the result of hardening is obtained. The membership degree of each sample point to the cluster is the value in the [0,1] interval, and each sample point belongs to only one class. Therefore, the algorithm easily falls into the local minimum value and is sensitive to the initial value. The PSO algorithm is a heuristic algorithm that was proposed by Kennedy and Eberhart and inspired by bird foraging behavior. It has the advantages of fast convergence and global optimization, so we combined it with the FCM algorithm to overcome the disadvantage of the FCM algorithm; it is called the FCAP algorithm.

In the FCAP algorithm, the key to the FCM algorithm is to determine the cluster center, and $x_{i}=(v_{i1},v_{i2},\cdots ,v_{ij},\cdots ,v_{ic})$ represents a cluster center set with one particle in the PSO, where $v_{ij}$ represents the *j*-th cluster center in the *i*-th clustering method. If the population size is N, there are N clustering methods.

The fitness level of each particle represents the quality of the clustering effect that is selected by this clustering center. For the evaluation of each particle, this article uses the following fitness function:

(4) $$f(x_{i})=J$$

The smaller that J is, the smaller the individual particle fitness is, so the clustering effect will be better.

According to the fitness value, the local position and the global position are calculated, and then the velocity and location of each particle are updated. Through the above steps, the FCAP algorithm can obtain a global approximate solution. The FCM algorithm is executed to obtain a global optimal solution again. In this study, we used the FCAP algorithm to complete the clustering of fog resources.

### 5.2. Resource Clustering Process Based on FCAP

In fog computing, we can divide the resource attributes into three categories: computation, storage, and bandwidth. Different types of tasks have different requirements for resources. For some computational tasks, computational resources are more important, while for some bandwidth tasks, bandwidth resources are needed. Therefore, in order to meet the requirements of different users, we first cluster the resources. Due to the dynamicity and heterogeneity of fog resources, it is difficult to accurately describe the attributes of individual resources. In this paper, we use the FCAP algorithm to cluster the resources according to the multidimensional attributes of the fog resources. The set of fog resources $R=\{r_{1},r_{2},r_{3},\cdots ,r_{m}\}$ indicates that there are m fog resource nodes, and each fog resource node contains n attributes, which can be expressed as follows:

(5) $$R=\left[\begin{array}{cccc} r_{11} & r_{12} & \cdots & r_{1n}\\ r_{21} & r_{22} & \cdots & r_{2n}\\ \vdots & \vdots & \vdots & \vdots \\ r_{m1} & r_{m2} & \cdots & r_{mn} \end{array}\right].$$

In Equation (5), $r_{ij}$ represents the *j*-th feature attribute of resource $r_{i}$.

**Figure 4:**
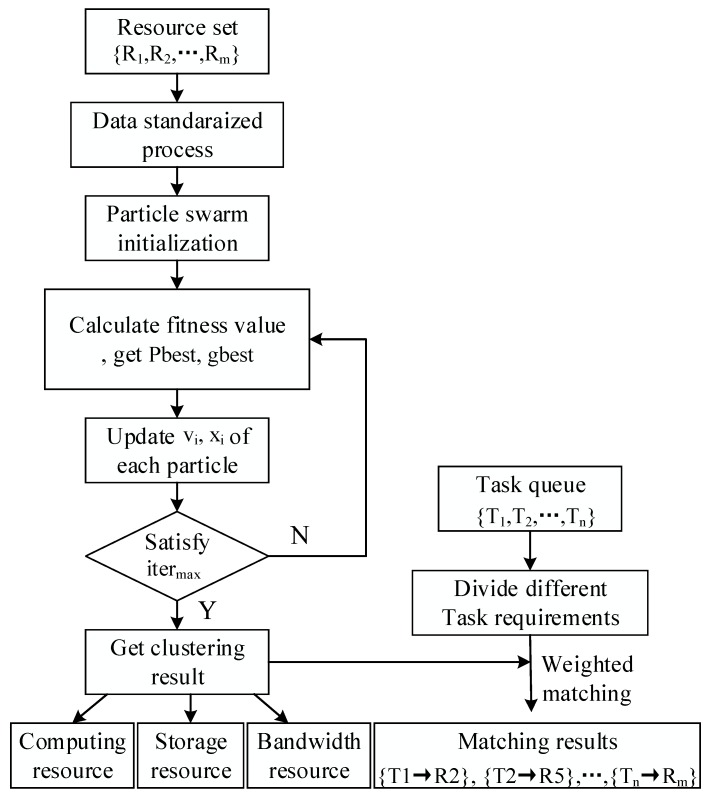
Resource clustering and scheduling process.

Figure 4 shows the resource clustering and scheduling process. Prior to the fuzzy clustering of resources, it is necessary to standardize and normalize the data for the various performance indicators of the resources. The steps for clustering fog resources are as follows.

(1) Data standardization

In a fog computing environment, due to the different dimensions of the characteristics of fog resources, if raw data are directly processed, the impact on the clustering results will be unbalanced. Therefore, in order to solve the adverse effects that are caused by this situation, first, the translation-standard deviation transformation is used to standardize the resource matrix data.

(6) $$r_{ij}^{\prime }=\frac{r_{ij}-\bar{r_{ij}}}{S_{j}}$$

(7) $${\bar{r}}_{ij}=\frac{1}{m}\sum_{j=1}^{n}r_{ij}$$

(8) $$S_{j}=\sqrt{\frac{1}{m}\sum_{i=1}^{n}{\left(r_{ij}-{\bar{r}}_{j}\right)}^{2}}$$

where the average value of each resource in the *j*-th dimension feature attribute is ${\bar{r}}_{j}$, and the standard deviation of each resource in the *j*-th dimension feature attribute is $S_{j}$. The processed data satisfy a standard normal distribution, which means that the mean is 0 and the standard deviation is 1.

(2) Data normalization

The standardized resource data cannot satisfy the planning of the fuzzy matrix. Therefore, translation-range conversion is used to convert the data in the matrix to a value in the interval of [0,1].

(9) $$r_{ij}^{\prime \prime }=\frac{r_{ij}^{\prime }-\min\{r_{ij}^{\prime }\}}{\max\{r_{ij}^{\prime }\}-\min\{r_{ij}^{\prime }\}}$$

where, $\min\{r_{ij}^{\prime }\}$ represents the minimum value in $\left\{r_{1j}^{\prime },r_{2j}^{\prime },\cdots ,r_{mj}^{\prime }\right\}$, and $\max\{r_{ij}^{\prime }\}$ represents the maximum value in $\left\{r_{1j}^{\prime },r_{2j}^{\prime },\cdots ,r_{mj}^{\prime }\right\}$.

(3) Fuzzy clustering of fog resources

The proposed fuzzy clustering algorithm based on particle swarm optimization is used to cluster the processed resource matrix data. The specific steps are as follows:

(i) Initialize the population of the particles, where each particle is a set of randomly generated cluster centers; divide the resources into three categories, and set the number of cluster centers as three.

(ii) Initialize the membership matrix $\mu_{ij}$, and calculate the cluster center $c_{j}$ according to Equation (10). The fitness function that is mentioned above is used to calculate the fitness value and determine the individual extreme value and the global extreme value. When the maximum number of iterations is satisfied, the iteration is stopped, and a set of cluster centers is obtained.

(10) $$c_{j}=\frac{\sum_{i=1}^{n}\mu_{ij}^{m}x_{i}}{\sum_{i=1}^{n}\mu_{ij}^{m}x_{i}}$$

(iii) The speed and the position of particles are continuously updated according to Equations (11) and (12). When the maximum number of iterations is satisfied, the iteration is stopped to obtain a set of cluster centers.

(11) $$v_{i}=\omega v_{i}+c_{1}r_{1}(pbest_{i}-x_{i})+c_{2}r_{2}(gbest_{i}-x_{i})$$

(12) $$x_{i}=x_{i}+v_{i}$$

where $x_{i}$ is the current position of the particle; $v_{i}$ is the velocity of particle; $c_{1}$ and $c_{2}$ are the accelerative constants; $pbest_{i}$ and $gbest_{i}$ are the local position and the global position of the particles, respectively; ω is the inertia weight, and $r_{1}$ and $r_{2}$ are randomly generated value between 0 and 1.

(iv) The above results are taken as the initial value of the FCM algorithm, and the FCM algorithm is executed to obtain a global optimal solution again.

(v) After the above clustering operations end, the fog resources are divided into three categories: computing resources, storage resources, and bandwidth resources.

### 5.3. Resource Scheduling Algorithm Design

After a reasonable division of fog resources, the resource scale in the scheduling process is reduced. User requirements can be divided into different classes. After finding the appropriate resource category, the user needs are matched with the resources in the class. To complete the resource scheduling, this paper uses simple weight matching to achieve it [13]. The weight matching formula is as follows.

(13) $$grade=\frac{\sum \left\|{req}_{i}-{res}_{i}\right\|\omega_{i}}{\sum \omega_{i}}$$

where $req_{i}$ represents the attribute of user’s needs, $res_{i}$ represents the attributes of the resources, and $\omega_{i}$ represents the weights of the attributes.

Different users have different resource requirements. For different task preferences, they can be divided into computing requirements, bandwidth requirements, and storage requirements. As with resources, each task also contains three attributes, each of which occupies a different weight. In the above formula, the attribute that is required by the user and the resource attribute are calculated together, and the highest score that is obtained is returned to the user as final result of the resource scheduling.

The pseudocode for the resource scheduling Algorithm 1 in this article is as follows:

**Algorithm 1**: resource scheduling algorithm based on FCAP(RSAF)**Input**: resource set $\left\{res_{1},res_{2},\ldots ,res_{m}\right\}$, task set $\left\{task_{1},task_{2},\ldots ,task_{n}\right\}$, m, ε, N **Output**: grade, matching result 1: original data processing 2: get the standardized matrix R, T 3: psofcm (N,K) →u and c 4: [r s]=size(R) 5: u=psofcm 6: **while**
t<M
**do** 7: t←t+1 8: $u^{\prime }\leftarrow u$ 9: calculate $u_{m}$ and v 10: **for**
k=1→K
**do** 11:  **for**
i=1→r
**do** 12:   distance(k,i)=Dist(data(i,:),v(k,:)) 13:  **end** 14: **end** 15: calculate $u^{\prime }$ and objfun 16: **if** norm$(u^{\prime }-u,\inf)<\varepsilon$
**then** 17:  break 18: **end if** 19: $u\leftarrow u^{\prime }$ 20: **end while** 21: get the three categories of resources 22: calculate grade($req_{i},res_{i},\omega_{i}$) 23: $grade=sum(norm(req_{i}-res_{i})*\omega_{i})/sum(\omega_{i})$ 24: **return** matching result $M:\{R_{i}\to T_{j}\}$

### 5.4. Algorithm Performance Analysis

The algorithm is divided into two phases. In the first phase, the particle population is initialized. Each particle in the population represents a set of arbitrarily generated cluster centers. Then, the membership matrix is calculated, and the fitness value is obtained. The velocity and position of the particles are updated to obtain the resource membership matrix constantly. Finally, the optimal resource membership matrix is obtained using the FCM algorithm. In the second phase, the weighted matching method is used to match the classified resource with the corresponding user request, and the final resource scheduling scheme is obtained and returned to the user. The FCM algorithm can easily be trapped in a local optimum, and is sensitive to the initial value. However, the PSO algorithm has the advantages of fast convergence and global optimization. Therefore, this paper combines the FCM algorithm and the PSO algorithm to complete the clustering of fog resources. We combine a fuzzy clustering algorithm with a heuristic algorithm to improve the resource clustering effect, which is better than a traditional clustering algorithm.

Theorem 4-1: the RSAF scheduling algorithm in this paper can accomplish resource clustering and scheduling in fog computing, and its time complexity is O(mnt). Here, m represents the number of fog resources, n represents the number of tasks, and t represents the maximum number of iterations.

Proof: from the analysis of the above algorithm, in the first stage of the cluster iteration process, it needs to go through t cycles, and the time complexity is related to the number of resources and the maximum number of iterations, which is O(mt). In the second stage of the scheduling process, the task needs to match its corresponding resources based on the first stage, and its time complexity is O(mnt). Therefore, the time complexity of this algorithm is O(mnt).

## 6. Experimental Results

To verify the effectiveness of the algorithm, the experimental platform of this paper uses MATLAB. The number of resource nodes and user requests are randomly set. The resource nodes are divided into three attributes in the experiment: computing power, bandwidth capability, and storage capability.

Table 2 shows the setting of each parameter in the experiment. Here, $c_{1}$ and $c_{2}$ represent the learning factors; $\omega_{\max}$ and $\omega_{\min}$ represent the maximum and minimum values of the inertia weight, respectively; m represents the fuzzy weighting coefficient; N represents the population size; and ε represents the convergence accuracy. In this paper, the inertia weight in the particle swarm optimization algorithm is linearly decremented according to Equation (14).

(14) $$\omega =\omega_{\max}-iter\times \frac{\omega_{\max}-\omega_{\min}}{iter_{\max}}$$

Here, iter and $iter_{\max}$ represent the current number of iterations and the maximum number of iterations, respectively.

The FCAP algorithm is used to cluster fog resources. The experiment uses the simulated resource dataset and the Iris dataset in the UCI machine learning database. In addition, we analyze and compare the two algorithms with respect to the convergence of the objective function.

**Figure 5:**
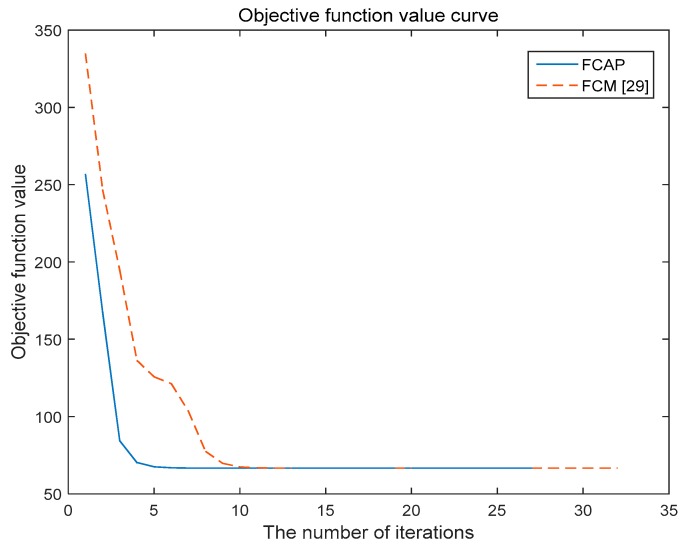
Objective function value curve for the Iris data set.

**Figure 6:**
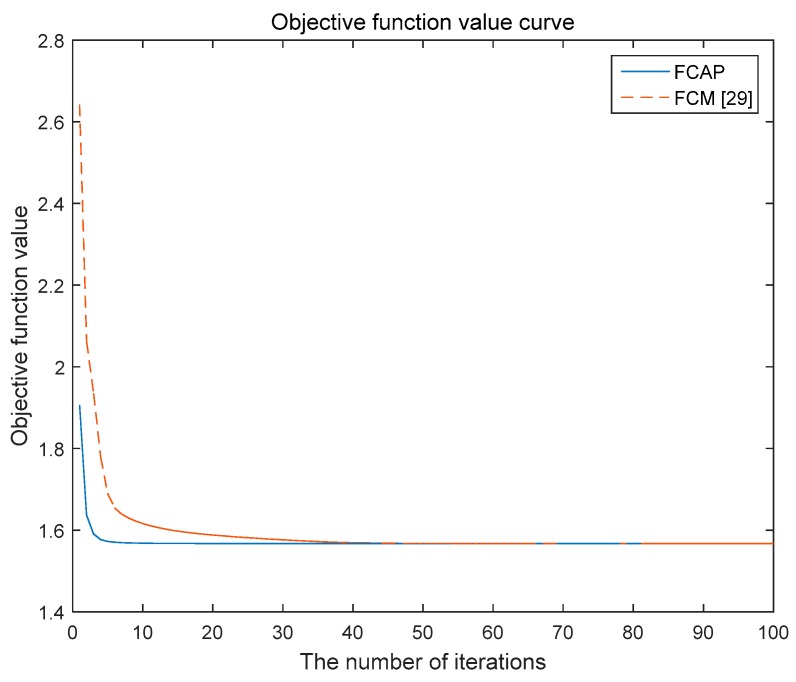
Objective function value curve for the simulated resource data set.

Figure 5 and Figure 6 show the variations of the objective functions of the traditional FCM algorithm and the FCAP algorithm with respect to the number of iterations in the case of two data sets. As seen from the above two figures, the FCAP algorithm had a faster convergence speed than the traditional FCM algorithm. The main reason is that the particle swarm optimization algorithm can find the cluster center faster and perform a global search to solve the problem that fuzzy clustering easily falls into the local minimum value. After reasonably dividing the fog resources, the scope of matching resources for users’ needs is reduced so that resource scheduling can be performed more efficiently.

To verify the accuracy of the clustering algorithm, we used the Iris and Wine datasets to analyze the performance of the algorithm. The two algorithms were run 20 times each, and the average values of the indicators were taken. The results are shown in Table 3.

As seen from the above Table 3, the FCAP algorithm has a higher clustering accuracy rate than the traditional FCM algorithm. Since the FCM clustering algorithm easily falls into the local minimum, the clustering effect is relatively poor. The FCAP algorithm can obtain the global optimal solution, and the clustering effect is relatively better. After correctly clustering the fog resources, the tasks can be matched with different types of resources, and, to some extent, the efficiency of resource scheduling is improved.

**Figure 7:**
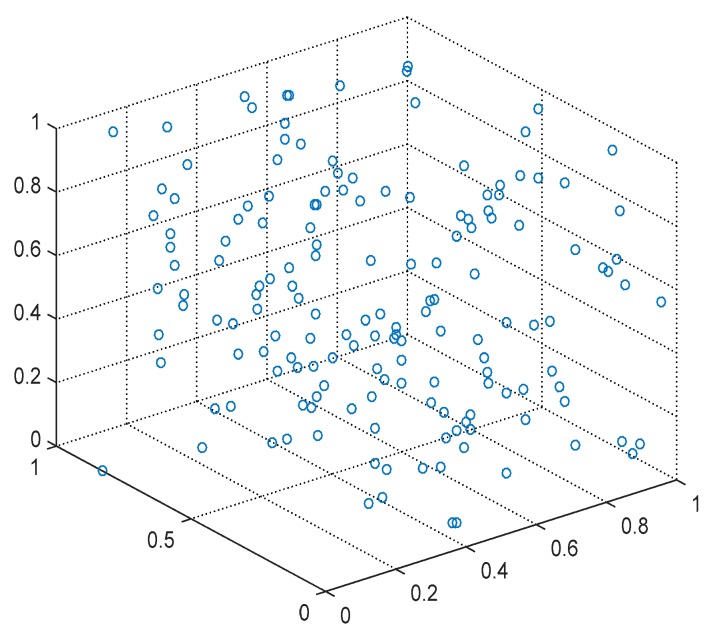
Original resources distribution.

**Figure 8:**
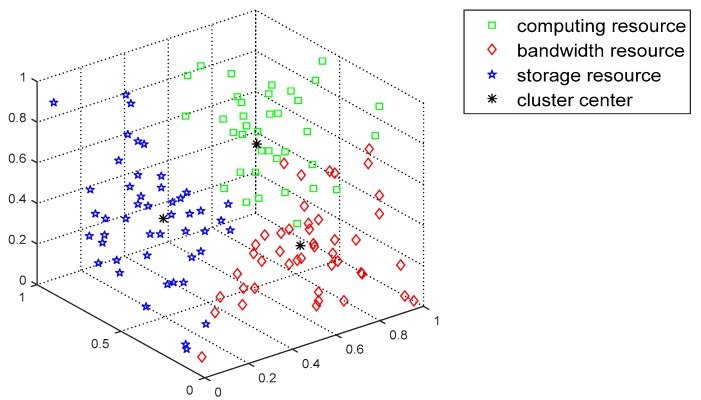
Resources distribution after clustering.

The FCAP algorithm is used to cluster fog resources. Figure 7 and Figure 8 show the distributions of the resources before and after clustering, respectively. From the graph, the FCAP algorithm can cluster the resources well, and, finally, the fog resources are clustered into three types of resources: computing, bandwidth, and storage resources. In addition, in order to better measure the matching of the task requests and resources, the numbers of users and resource nodes that are randomly generated are 10 and 20, respectively.

In this experiment, the user requirements were divided into computing requirements, bandwidth requirements, and storage requirements according to the different needs. Different types of user needs will be selected to match the different categories of resources, and the final matching results will be returned to users. Finally, the final scheduling scheme is shown in Table 4.

To evaluate the rationality of resource scheduling, we calculated the user satisfaction index. This paper refers to the user satisfaction calculation method in [12], and defines the formula for calculating user satisfaction as follows:

(15) $$\bar{U_{sat}}=\ln(\frac{\alpha (t_{comp})}{r_{comp}}+\frac{\beta (t_{netw})}{r_{netw}}+\frac{\gamma (t_{stor})}{r_{stor}})$$

where $t_{comp}$, $t_{netw}$ and $t_{stor}$ represent the requirements of the task for the resource calculation attribute, bandwidth attribute, and storage attribute, respectively. α, β and γ represent the empirical coefficients of the computing, bandwidth, and storage resource requirements, respectively. In addition, $r_{comp}$, $r_{netw}$ and $r_{stor}$ represent the calculation attribute, bandwidth attribute, and storage attribute of the resources that match the task, respectively.

**Figure 9:**
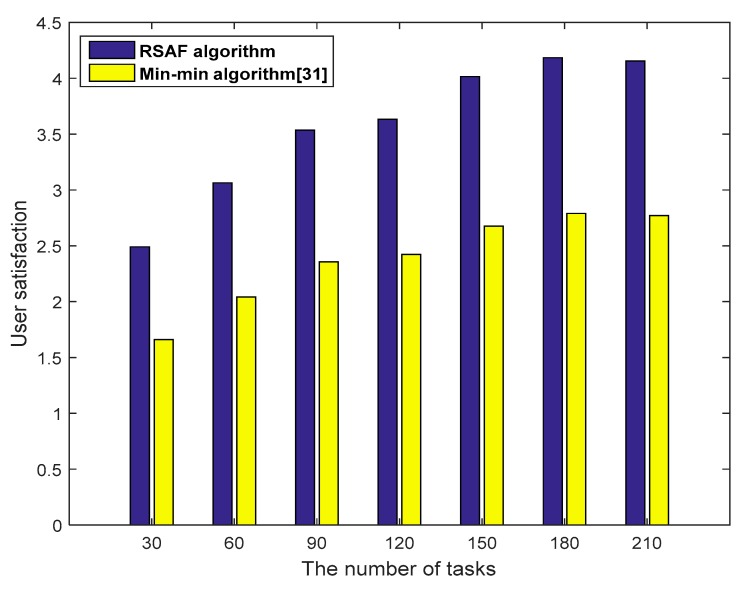
Comparison of user satisfaction.

Figure 9 shows the comparison of the user satisfaction of the proposed RSAF algorithm and the Min-min algorithm [31]. The Min-min algorithm allocates the shortest tasks to the fastest processing resources to ensure that the overall task completion time is the shortest. However, it is easy to cause a load imbalance, resulting in lower user satisfaction. As seen from the above figure, compared with the Min-min algorithm, the RSAF algorithm can achieve a reasonable matching of user requests and fog resources and ensure better user satisfaction.

## 7. Summary and Future Work

This paper studied the resource scheduling problem in fog computing. First, we applied the FCAP algorithm to cluster fog resources, which narrows the range of user requirements for matching resources. We also propose the RSAF algorithm to accomplish resource scheduling. Finally, from the experimental analysis, the objective function value of the FCAP algorithm in this paper was shown to have a faster convergence speed than the FCM algorithm. In addition, the proposed RSAF algorithm can match user requests with the appropriate resource categories quicker and improve user satisfaction. In future work, we will consider the dynamic changes of resources and propose a new scheduling strategy to improve the utilization of resources and ensure user satisfaction.

## Figures and Tables

**Table 1:** Differences between cloud-based scheduling and fog-based scheduling.

	Cloud-Based Scheduling	Fog-Based Scheduling
**Advantages**	Strong computation power	Low delay and location awareness
	Apply to processing delay- tolerant user requests	Apply to processing delay-sensitive user requests
**Disadvantages**	Consume a lot of bandwidth	Weaker computation power
	A large transmission delay	Not applicable to processing large-scale requests

**Table 2:** Experimental parameter settings.

Parameter	Value
$c_{1}$	1.2
$c_{2}$	1.2
$\omega_{\max}$	0.9
$\omega_{\min}$	0.4
m	2
N	50
ε	1.0 × 10^−6^

**Table 3:** Comparison of the clustering accuracy.

Algorithm	Data Set	Correct Clustering Sample Number	Error Clustering Sample Number	Correct Rate
FCM	Iris	134	16	89.3
	Wine	122	56	68.5
FCAP	Iris	138	12	92
	Wine	129	49	72.5

**Table 4:** User requirements and resources matching results.

Resource Classification	Scheduling Results
$\left\{r_{3},r_{4},r_{7},r_{9},r_{16},r_{17},r_{19}\right\}$	$\{t_{1}\to r_{4}\},\{t_{6}\to r_{9}\},$ $\left\{t_{9}\to r_{17}\right\}$
$\left\{r_{2},r_{8},r_{10},r_{12},r_{14},r_{18},r_{20}\right\}$	$\{t_{2}\to r_{12}\},\{t_{8}\to r_{14}\},$ $\left\{t_{10}\to r_{2}\right\}$
$\left\{r_{1},r_{5},r_{6},r_{11},r_{13},r_{15}\right\}$	$\{t_{3}\to r_{15}\},\{t_{4}\to r_{5}\},$ $\left\{t_{5}\to r_{11}\},\{t_{7}\to r_{1}\right\}$
